# Is urinary kidney injury molecule-1 a good marker for acute kidney injury in septic shock?

**DOI:** 10.1186/cc11746

**Published:** 2012-11-14

**Authors:** J Göschl, L Sudhoff, M Koehler, W Rachinger, J Briegel, I Kaufmann

**Affiliations:** 1 Ludwig-Maximilians-University of Munich, Germany

## Background

Kidney injury molecule-1 (KIM-1) is a new biomarker promising a better detection and diagnostic quality of acute kidney injury (AKI).

## Methods

This clinical prospective study - approved by the local ethics committee - was performed to assess urinary levels of KIM-1 by ELISA technique in 38 patients with septic shock (Table 1). They were prospectively enrolled within 24 hours of onset of signs of infection, if they met the criteria for septic shock as defined by the members of the ACCP/SCCM Consensus Conference Committee. KIM-1 levels were quantified on admission (baseline, day 0) and on days 4, 7 and 10 of the ICU stay. The patients were classified by AKIN and RIFLE criteria. Data were analyzed with regard to the prognostic value of survival, need for renal replacement therapy (RRT) and correlation with renal function parameters (plasmatic urea and creatinine, creatinine elimination and clearance, free water clearance, fractional sodium excretion) or hepatic laboratory findings (albumin, total bilirubin, ASAT, ALAT, γ-GT). Data are given as mean ± SEM.

**Table 1:** Demographic data

Age (years)	57.2 ± 2.6
Gender (male/female)	27/11
BMI (kg/m^2^)	29.7 ± 1.5
Length of ICU stay (days)	19.5 ± 2.7
Mortality (%)	13.2 (5 out of 38 patients)
SAPS score	63.4 ± 2.7
APACHE II score	28.8 ± 1.3
SOFA score	12.7 ± 0.5
MOD score	10.4 ± 0.6
RIFLE (number per group)	no AKI = 28; risk = 4; injury = 3; failure = 3;
AKIN (number per group)	no AKI = 25; stage 1 = 1; stage 2 = 1; stage 3 = 11
Need for RRT (yes/no)	16/22

## Results

**Figure 1:**
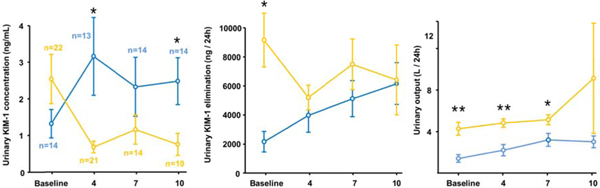
**Urinary KIM-1 concentration (A) and elimination (B) in comparison with urinary output (C) per 24 hours in patients with (blue line) and without (yellow line) RRT at baseline and on days 4, 7 and 10**. *P < 0.05, **P < 0.001.

The urinary KIM-1 concentration on admission showed no significant difference with respect to survival of patients. However, the urinary KIM-1 concentration determined on days 4, 7 and 10 was higher in patients surviving septic shock (*P *< 0.001 on days 4 and 7). Elimination displayed lower levels in deceased patients (*P *< 0.05 on days 0 and 4), whereas urinary output was higher in survivors during the whole ICU stay (*P *< 0.05 on day 7). Urinary KIM-1 concentration did not differ between AKIN and RIFLE classification groups. Urinary KIM-1 elimination per 24 hours on days 0, 4 and 7 was higher in stage 1 than in stage 2 or 3 of the AKIN classification, respectively (both *P *< 0.05). If patients were classified by RIFLE criteria, urinary KIM-1 elimination was also higher in the risk group as compared with the injury or failure group without reaching significance. However, the need for RRT was reflected by a higher urinary KIM-1 concentration after admission (*P *< 0.05 on days 4 and 10), a lower KIM-1 elimination and urinary output during the whole ICU stay (KIM-1 elimination: *P *< 0.05 on day 0; urinary output: *P *< 0.001 on days 0 and 4, *P *< 0.05 on day 7; Figure 1A,B,C). Neither urinary KIM-1 concentration or elimination correlated with any renal and hepatic function parameter.

## Conclusion

Urinary KIM-1 can be used as a prognostic factor for survival and need for RRT in septic shock patients.

